# Measuring Intimate Partner Violence and Traumatic Affect: Development of VITA, an Italian Scale

**DOI:** 10.3389/fpsyg.2018.01282

**Published:** 2018-07-26

**Authors:** Gina Troisi

**Affiliations:** Department of Humanities, University of Naples Federico II, Naples, Italy

**Keywords:** intimate partner violence, shame, guilt, terror, fear, psychodynamic perspective, women’s health

## Abstract

In a global context where the percentage of women who are victim of violence is still high (World Health Organization, 2013), intimate partner violence (IPV) can be considered the most widespread form of violence against women: in such cases violent attacks are perpetuated or threatened by a partner or ex-partner within an intimate relationship, which makes its recognition more difficult. IPV requires specific tools and, although the literature has highlighted the specific role played by some emotions (such as shame, guilt, and fear) that keep women experiencing this violence in a state of passivity and confusion, to date too little attention has been given to the construction of sound instruments able to detect post-traumatic affectivity. Such instruments could facilitate women who have suffered from IPV in recognizing it and in making the responses of women’s health services more sensitive and structured. This study illustrates a sequential item development process to elaborate a new self-report instrument (VITA Scale: Intimate Violence and Traumatic Affects Scale) for assessing the intensity of post-traumatic affect derived from IPV. Within a psychodynamic perspective, the scale is characterized by four affects: fear, as a state of alarm elicited by the avoidance of the danger; terror, as a paralyzing state that hinders an active process of reaction; shame as a strong exposure to the other that disarms the individual and the guilt as a defensive dimension aiming at the restoring of the link with the abusive partner. Trough specific methodological steps, a 28-item set was selected and administered to a sample of 302 Italian women who declared themselves as having suffered from IPV. Explorative and confirmatory factor analysis, as well as correlations with well-established concurrent tools were computed in order to investigate its psychometric property. A factorial structure composed of four factors, consistent with theoretical scales and a good internal consistency (Cronbach’s alphas from 0.80 to 0.90) emerged. The VITA Scale could be a useful tool for clinicians and researchers to investigate the intensity of the affective state of the woman suffered from IPV. It could be useful to better address the clinical practice and therapeutic intervention planning.

## Introduction

### Intimate Partner Violence

With an estimated global prevalence of 30% (World Health Organization, 2013), intimate partner violence (IPV), can be considered the most widespread form of violence against women.

According to the definition of the American Psychological Association and Presidential Task Force on Violence and the Family (1996), IPV, is the physical, sexual, psychological, economical or stalking abuse, both concrete and menaced, perpetuated by current or ex-partners. In the European Union Member States the 22% of women have suffered from physical and/or sexual violence by partners since the age of 15, with a prevalence across countries ranging from 13 to 32% (European Union Agency for Fundamental Rights, 2014). In Italy, according to a national survey by the National Institute of Statistics (ISTAT, 2015), two million eight hundred thousand women between 16 and 70 years have experienced at least one episode of sexual or physical violence by partner or ex-partner. Indeed current or ex-partners commit the most serious violence and are involved in 62.7% of rapes. IPV can include sexual assault: according of World Health Organization, Department of Injuries and Violence Prevention (2002) one in four women experiences sexual violence by her intimate partner. On the other hand, sexual harassment, such as sex-related verbal or physical behavior that is annoying or disrespectful to the person who suffers it Rubinstein (1987) and Piotrkowski and Brannen (2002) is perpetrated more frequently in the work environment by colleagues or employers. IPV and sexual harassment have many similarities: they are both mainly crimes against women by known perpetrators, and occur in places perceived as safe by victims, like the home or the workplace (Lawson, 2012).

Although it cannot be viewed as a unidirectional phenomenon, IPV concerns a higher percentage of violence of the man against the woman (World Health Organization, 2013). Furthermore, according to World Health Organization (2013) even if IPV can occur against men, men injured by their partners had high rates of IPV perpetration themselves and the violence carried out by women may often present itself as self-defense.

Initially, this phenomenon was investigated within Feminist Movements. In this perspective, IPV was linked to male dominance, rooted in the patriarchal traditions of heterosexual relationships, expressed through control and power dynamics (Dobash and Dobash, 1979; Pence and Paymar’s, 1993; Ferraro, 1997; Campbell et al., 1998). According to a recent overview (Bell and Naugle, 2008) the “Feminist Theory,” and the “Power Theory” constitute the *Sociocultural theories*, that derive the roots of violence not only from culture but also from the family structure (Straus, 1976).

On the other hand *Individual Theories*, include the “Social learning theory,” the “Background/situational model” (Riggs and O’Leary, 1996) and the “Personality/typology theories” that bring the origins of violent conduct back to behaviors learned during childhood (Mihalic and Elliott, 1997; Shook et al., 2000), or to situational factors or elements linked to individual background or, again, to personal characteristics of victims and perpetrators (Koss et al., 1994).

These two classifications were mentioned to explain the complexity of the phenomenon of IPV, whose origins can be traced both at a sociocultural level and in the relationship dynamics of the specific couple.

Other studies focused on the descriptive factors of different types of IPV. Johnson (1995) has distinguished two forms of male violence against female partners: *intimate terrorism* and *situational couple violence.* This distinction may be important in planning prevention and intervention programs and to understand the specific consequences that these two forms of violence can have at the psychic level. In *intimate terrorism* the perpetrator imposes strict control on the partner, through emotional abuse, using children, isolation, threats, intimidation, economic abuse, and blaming. On the other hand, *situational couple violence* concerns a certain altercation that turns into an unstoppable series of escalating violence but with no evidence of the perpetrator exerting control over the partner (Kelly and Johnson, 2008). It is most likely to be described within the conceptual framework of family conflict theory (Straus and Gelles, 1990; Bradbury et al., 2001). Intimate terrorism is probably best conceptualized through the patriarchal pattern of male dominance (Frieze and Browne, 1989). This violence is rarely an isolated incident, as it often turns into more severe episodes of violence in an escalation (Walker, 1977; Coleman, 1997), which may have dangerous consequences for the partner’s physical, psychological, and social well-being.

Several studies suggest that depression, panic attacks, inability to cope, suicide attempts, non-suicidal self- injury, post-traumatic stress disorder (PTSD), and alcohol or drugs abuse may be some possible consequences of IPV on the health of the victims (Campbell, 2002; Ellsberg et al., 2008; Pico-Alfonso et al., 2008; Gargiulo et al., 2014). However, few studies in this field have underscored the role of the subjective affective experience of victimization. The different forms in which the IPV can manifest itself within the couple can even result in different affective reactions (Jaquier and Sullivan, 2014).

Psychological violence is always present where there is any other form of violence within a romantic relationship and it is identified as their main source of distress by women who have suffered from IPV (Murphy and O’Leary, 1989; Ronfeldt et al., 1998; Hamby and Sugarman, 1999). IPV, in its form of *Intimate terrorism* can be conceptualized as a sort of private dictatorship that is developed through progressive and disguised attacks. The implicit aim of the abuser is to deprive the victim of his/her individuality, destroy his/her subjectivity, by imposing strict control and exerting physical and psychological violence in order to make the victim a powerless object at the mercy of the dominant partner. The affect of terror seems to play a major role. When the violence appears to be isolated and not restricted to a relationship that assumes the characteristics of private dictatorship, the affect of fear is more likely to be present with the behavioral reaction that follows. The victim would be forced to escape in anguish or, alternately, to react with anger and attack (Nunziante Cesàro and Troisi, 2016).

The subjective affective experience of the victim of violence plays a relevant role in the maintaining the violent relationship (Herman-Lewis, 1992; Hirigoyen, 2005).

Many studies testified how IPV is more difficult to be recognized both from the victims’ perspective (Herman-Lewis, 1992; Hirigoyen, 2005; Reale, 2011) and from the society perspective (Romito, 2005; Arcidiacono and Di Napoli, 2012).

Few studies focused on the reasons for exiting or remaining in the violent relationship (Bell and Naugle, 2005).

Several authors showed that the silence of victims of IPV and their ability of carry out help seeking strategies can be influenced by a combination of different factors (Tjaden and Thoennes, 1998; Rennison and Welchans, 2000) Together with cognitive, social and psychosocial factors, emotional factors, such as emotional dependence, fear, guilt and shame, play a main role in maintaining of abusive relationship (Tjaden and Thoennes, 2000; Margherita and Troisi, 2014).

This study focuses on emotional factors maintaining the violent relationship in particular guilt, shame, fear, and terror.

### Affect and Trauma in Intimate Partner Violence

Trauma is the main consequence of IPV (Resnick et al., 1993; O’Keefe, 1998; Ehrensaft, 2009). If the traumatic events have occurred repeatedly or chronically, complex PTSD is diagnosed (Herman-Lewis, 1992). This involves specific alterations in affect regulation.

Several studies remark on the importance of emotion dysregulation in PTSD (Van der Kolk, 1996; Cloitre, 1998) since it leads to a lack of awareness of the emotional states the trauma may induce (Litz et al., 2000; Bouton et al., 2001; Hunt and Evans, 2004; Orsillo et al., 2004; Veazey et al., 2004). Through the negative effect on interpersonal relationships and on an individual’s overall functional capacity, emotion dysregulation may have an impact on the maintenance of PTS symptoms (Cloitre et al., 2002). Particularly, negative emotions were important for understanding the PTSD (Dalgleish and Power, 2004; Resick and Miller, 2009). Shame and guilt contribute to the development and maintenance of PTSD (Lee et al., 2001; Wilson et al., 2006).

Few empirical studies have addressed the specific relation between emotions and PTSD in IPV.

In traumatic experiences such as sexual/physical abuse perpetrated by a known and/or trusted perpetrator, heightened levels of shame compared to fear that would probably accompany a trauma characterized by physical threat, in particular among women (Andrews et al., 2000).

In this study guilt, shame, fear, and terror are considered “affects” within a metapsychological and psychoanalytic framework.

“Affects” were defined as a range of emotions, feelings and passions, which could be represented by a metaphorical image (Green, 1973; Imbasciati, 1991).

Psychoanalytic theories on the trauma suggest how it leads to the collapse of the construction of the meanings processes (Bohleber, 2007; Levine, 2014) and how it disrupts the capacity for representation of mentalization (Levine, 2014).

In literature little attention has been devoted to the affect of shame in interpersonal violence.

In victims of violence, the sense of passivity and helplessness and the feeling of being treated as an object could be traced back to the affect of shame, understood as something that makes the victim feel exposed, naked at the mercy of the other, who, as in the primary impotence at the origin of the life, has the power of life and death over the subject (Margherita and Troisi, 2014). A masking of shame through guilt can more easily permit forgiveness, through a reparative gesture, assuring the maintenance of the link with the partner and restoring an active position in the relationship by taking responsibility for other’s behavior. This could explain why self-blaming and silence are such widespread phenomena in IPV (Margherita et al., 2014).

More recently the psychodynamic of affects is moving toward a more precise differentiation between guilt and shame (see, for example Tisseron, 1992; Morrison, 1999; Tangney and Dearing, 2002; Ciccone and Ferrant, 2015). Shame was conceptualized as an archaic and destructive affect that draws the individual’s primary impotence and puts a narcissistic failure at stake. Shame accompanies the perception of a failure and the Self is placed in a passive state, where hiding prevails (Morrison, 1999).

In contrast, guilt may be associated with transgression and the Self remains active, absorbed in the action, even during the repair (Tisseron, 1992).

A previous qualitative study allowed an in-depth analysis of the role played by the affects of fear, shame, and guilt in women victims of IPV (Nunziante Cesàro and Troisi, 2016). Authors underscored the difference between fear, associated with the escape from danger and therefore understood as an active defense, and terror associated with paralysis and freezing, in line with psychoanalytical (Diel, 1956; Clit, 2002) and neurophysiological studies (Hagenaars et al., 2014). Considering the three possible reactions that an individual can develop in the face of danger, the attack is associated with anger, the escape is associated with fear and abandonment is associated with terror. Fear, therefore, seems to be a protection that puts the subject in a state of activity and makes them alert, activating sensorial and perceptive systems linked to the awareness of an event that is perceived as traumatic (Nunziante Cesàro and Troisi, 2016).

It would be inappropriate to involve the affect of fear, instead, in situations of private dictatorship because it presupposes an actual danger and foresees a peculiar reactivity on both the behavioral and psychic level.

The situations of extreme violence crystallize the experiences of archaic terror, re-actualize the proven perceptions and the defenses used by the subject (Nunziante Cesàro and Troisi, 2016).

### Measuring the Traumatic Affect in Intimate Partner Violence

Affectivity involved in IPV requires valid and specific tools to be measured for quality and quantity. Among existing screening instruments used for the identification of women victims of IPV as the Index of Spouse Abuse (ISA; Hudson and McIntosh, 1981), the Abuse Risk Inventory (ARI; Yegidis, 1989), the Composite Abuse Scale (CAS; Hegarty et al., 1999), or the Conflict Tactics Scale (CTS; Straus, 2017) should be mentioned. However, these screening tools seem to take into account all possible forms of violence. More importantly, to our knowledge (Rabin et al., 2009) a comprehensive examination of their psychometric properties is lacking. There are also several validated IPV risk assessment instruments, for example Danger Assessment (DA; Campbell, 2004) to assess risk factors for Intimate Partner Femicide, the Ontario Domestic Risk Assessment (ODARA; Hilton et al., 2004) and Spousal Assault Risk Assessment (SARA; Kropp and Hart, 2000; Baldry, 2006).

However, with the aim of measuring the consequences of IPV, several, non-IPV specific tools are used, and the distress is often just evaluated asking the women to assess their general mood.

Exemples of tools used for symptom detection, or multidimensional self-report symptom inventories include: Symptom Checklist-90-R (SCL – 90; Derogatis and Cleary, 1977), or scales that investigate specific dimensions such as depression, using by Beck Depression Inventory (BDI; Beck, 1961), or PTSD tools like The Post-traumatic Stress Disorder Checklist (PCL; Weathers et al., 1991) or the Peritraumatic Dissociative Experiences Questionnaire (PDEQ; Marmar et al., 1997) or the Peritraumatic Distress Inventory (PDI; Brunet et al., 2001) or scales that measure psychological well-being, such as the Psychological General Well-Being Index (PGWBI; Dupuy, 1984), or the quality of life, such as Quality of life (QOL; Flanagan, 1978) or on resilience, such as Resilience Scale for Adult (RSA; Friborg et al., 2003).

However, no tools were developed to measure the traumatic impact that IPV can generate on the affective world of women, hence the necessary importance to the level of emotional abuse that accompanies victimization is not considered (Jaquier and Sullivan, 2014).

The development of a valid and reliable scale could aim at measuring the post-traumatic affectivity in situations of IPV, facilitating the identification and the therapeutic process of women victims of IPV, as well as making the responses of health services more sensitive and structured. This study illustrates methodological steps aimed at the development of a self-report instrument for identifying the post-traumatic affectivity in women who have suffered from IPV.

## Materials and Methods

### Participants

The sample comprised 302 Italian women (*M*: 30.63; *SD*: 18.5 years) recruited online, through mailing lists and social networks. The whole sample was split randomly into two congruous subsamples (subsamples A and B) for the analysis of its factor structure (Bollen, 1989).

The two sub-samples did not differ significantly in age (*t*_290_ = 1.39, *p* = 0.164) marital status (*t*_300_ = 0.124, *p* = 0.901), nor awareness of violence (*t*_217_ = 1.94, *p* = 0.06) and period of violence (*t*_217_ = 1.58, *p* = 0.116).

Subjects included in the whole sample were mostly unmarried (81.4%) while 12.6% were married, 1.9% divorced, 3.5% separated and 0.6% through a divorce.

Regarding sexual orientation, 87.1% stated that they were heterosexual, 6% bisexual, 2.2% homosexual. The study participants mostly had a high level of education: 31.3% had a master’s degree and 20.2% a 3-year degree, 16.7% a post-graduate degree and 30% a high-school diploma. As regards the employment, 34.4% of the entire sample were students, 25.5% were self-employed workers, 15.5% were employed, 5.4% was made up of women without any employment and the 1.9% of the sample were managers.

At the time of the compilation of the questions, 71.3% of the subjects were involved in a current romantic relationship, while 28.4% were single. Moreover, 84.5% of women had no children and the remaining had from one to three offspring.

### Measures

#### Intimate Violence and Traumatic Affect Scale (VITA Scale) (Troisi, 2017)

The original version of the VITA is an Italian 28-item self-report measure used to assess the intensity to affects in women that suffered from IPV. Of all the items, 5 were related to the affect of *fear*, 7 belonged to the affect of *terror*, 10 were related to *shame* and 6 to *guilt*. Items are rated on a Likert type scale (from 1 = *never to* 5 = *often*). In the present study, the Cronbach’s alpha was 0.79 for Fear, 0.9 for Terror, 0.93 for Shame, 0.87 for Guilt and 0.93 for the total scale. The process of the development of VITA Scale is described below.

#### Intimate Partner Violence Check List

The checklist was obtained from the National Association DiRe “Networking of Women against violence,” the first Italian association of non-institutional anti-violence centers. The descriptions are set up in the form of questions rated on five-point Likert type scale (from 1 = *never* to 5 = *always*). Five forms of violence are included: *psychological violence* (18 items) containing every form of abuse that damages the identity of the victim; *sexual violence* (3 items) including the imposition of sexual practices or sexual relationships that cause physical harm obtained through threats of various kinds; *physical violence* (7 items) including the use of any act guided by the intention to do evil or to terrorize the woman who has suffered violence; *stalking* (8 items) including controlling behavior performed by the persecutor, *economic violence* (6 items) as a form of direct control, which limits the victim’s economic independence. This checklist was used for descriptive purposes to identify what kind of violence the study participants suffered.

#### Questions About Violence

Further questions on the awareness of violence (*Have you ever suffered any form of violence?*), on the period of her life in which the violence had been experienced, on the author of the violence, on the intensity of suffered violence (if isolated or repeated).

#### Questions About Help Seeking

These questions aimed at identifying the presence or absence of reporting and a help seeking process, and if present the type of help requested (informal and formal), if absent the reasons behind the failure to request help.

#### Difficulties in Emotion Regulation Strategies (DERS) (Gratz and Roemer, 2004)

It is a 36-item multidimensional self-report measure of difficulties in emotion regulation. Items are assessed on a five-point Likert scale ordered from 1 = *almost never* to 5 = *almost always*. The DERS assesses difficulties in six clinically relevant dimensions of emotion regulation: (a) non-acceptance of emotional (*Non-acceptance)*, (b) difficulty engaging in goal-directed behavior in distress situations (*Goals*), (c) inability to control behavior when distressed (*Impulse*), (d) lack of awareness of emotions (A*wareness)*, (e) limited access to strategies that are perceived as effective for emotion regulation, and (f) lack of emotional clarity. The DERS showed adequate construct and predictive validity, as well as good test–retest reliability (ρI = 0.88; Gratz and Roemer, 2004). Also, the Italian adaptation it showed good psychometric properties (Giromini et al., 2012). In the present study, internal consistencies for the total and subscale scores were good, ranging from 0.81 (for A*wareness*) to 0.89 (for *Non-acceptance*).

#### Impact of Event Scale (IES) (Horowitz et al., 1979)

It is a self-report measure composed of 15 items on a four-point Likert scale (ranging from 1 = *not at all* to 4 = *often*). The IES tapped two-specific answers to traumatic events: (a) *intrusion*, intended as emerging of undesired ideas, images, feelings, or dreams that remind to the event and (b) *avoidance*, intended as elusion of certain ideas, feelings, or situations linked to the stressful situation. In its Italian version Cronbach’s alphas were 0.84 for the intrusion subscale and 0.71 for the avoidance subscale (Pietrantonio et al., 2003). In the present study, the IES total score yielded Cronbach’s alpha of 0.93, 0.92 for the *intrusion* subscale and 0.89 for the *avoidance* subscale.

#### Other as Shamer Scale (OAS) (Goss et al., 1994)

It includes 18 items to measure the external shame, as a global judgment about how the self is evaluated by others. Items are rated on a five-point Likert-type scale (ranging from 0 = *never*to 4 = *almost always*). It was made up of three subscales: (a) *inferiority*, related to being seen as inferior; (b) *emptiness*, related to being seen as empty; and (c) *mistake*, related to how others are vigilant to mistakes one makes (Goss et al., 1994). In the Italian version of OAS, the Cronbach’s alphas were 0.87 (Balsamo et al., 2015c; Saggino et al., 2017).

In the present study, the OAS yielded Cronbach’s alpha of 0.94 for the total score and 0.92 for the *Inferiority*, 0.83 for *Emptiness*, and 0.86 for *Mistake* subscale.

#### Coping Orientation to Problems Experienced 25 (COPE-NVI-25) (Foà et al., 2015)

This 25-item scale is a reduced form of the Coping Orientation to Problem Experienced (COPE-NVI) developed by Carver et al. (1989). Items ask to assess how often the subject implements a certain coping process in difficult or stressful situations. The selected subscales measure: *Avoidance Strategies* (5 items), which concern the negation and natural detachment; *Transcendent Orientation* (4 items); *Positive Attitude* (6 items); *Social Support* (5 items) related to the search for understanding, information and of emotional outpouring; *Problem Orientation* (5 items) related to the use of active planning strategies and suppression of alternative activities. The Cronbach’s alpha was 0.70 for all dimensions, excepting for *avoidance strategies*, which nevertheless presents values considered satisfactory (Sica et al., 2008). In our sample, for the COPE-NVI 25 total score Cronbach α is equal to 0.85. For the different subscales, it was 0.76 for *avoidance strategies* 0.96 for *transcendent orientation* = 0.80 for *positive attitude*, 0.94 for *social support* and 0.83 for *orientation to the problem*.

#### Teate Depression Inventory (TDI) (Balsamo and Saggino, 2014; Balsamo et al., 2014)

It is a 21-item self-report tool that aims to measure depressive symptoms as described by the latest editions of the Diagnostic and Statistical Manual of Mental Disorders (DSM; American Psychiatric Association, 2013) on a five-point Likert-type scale (ranging from 0 = *always* to 4 = *never*). It was developed via Rasch logistic analysis of responses (Rasch, 1960), within the framework of Item Response Theory, in order to overcome psychometric weaknesses of existing measures of depression (Balsamo and Saggino, 2007). Recent literature suggested that TDI demonstrate good psychometric properties (Balsamo et al., 2013a,c, 2015a,b; Innamorati et al., 2013; Carlucci et al., 2018; Contardi et al., 2018; Saggino et al., 2018). In the present sample, Cronbach’s α was 0.95.

### Procedure

#### Questionnaire Development

##### Generation of the preliminary item list

In a qualitative study, a group of 10 women (age *M*: 42.25; *SD*: 4.9 years) who had suffered from IPV and who had sought help services, were interviewed. Affects of guilt, shame, fear, and terror were identified and explored as associated with the situation of violence suffered by the women (Nunziante Cesàro and Troisi, 2016). A pilot study was carried out, through an online *ad hoc* questionnaire developed, in order to test this method of administration. The on-line administration appeared to be more appropriate for recruiting participants who did not want to access the help services, because guaranteed them protection and respect for their own privacy. Furthermore, the pilot study allowed investigation of the means of expression, the sequencing rule for the questions and the types of IPV suffered and to evaluate the response format (Troisi, 2017).

A qualitative selection of the pool of items was carried out on the basis of the words used by the women in the qualitative study, the results from the pilot study and the theoretical assumption. Items were expressed in the metaphorical form. Typically, the language of affects can be more readily evoked by the use of metaphor, often linked to a shared collective symbolization (Imbasciati, 1991; Tisseron, 1992). Therefore, items have been organized through their insertion into different areas related to the following affects: fear, terror, shame, and guilt.

Within the semantic area related to the fear, fear was considered as a state of alarm elicited by the avoidance of the danger (Diel, 1956; Hagenaars et al., 2014). Within the semantic area of the terror, this affect was framed as a paralyzing state that hinders an active process of reaction to danger (Clit, 2002; Nunziante Cesàro and Troisi, 2016). The semantic area of the shame defines it as a strong exposure to the other that disarmed the individual and makes him animated by a sense of failure and passivity (Tisseron, 1992; Lewis, 1995; Pandolfi, 2002; Ciccone and Ferrant, 2015). The semantic area of the guilt focused on its defensive dimension aimed at the restoration of the relationship with the partner, assuming responsibility for the violence suffered and taking an active position in the relationship (Tisseron, 1992; Pandolfi, 2002; Ciccone and Ferrant, 2015).

As a strategy for developing items useful for capturing the meaning of the psychological constructs of affects here defined, three experts, of whom one psychotherapist/researcher and two clinical psychologists, were asked independently to assess items on a Likert type scale. The item pool generated by these procedures comprised 30 items, including 6 for the semantic area of fear, 7 for the semantic area of terror, 10 for the semantic area of shame and 7 for the semantic area of guilt. A five-point Likert-type scale raking from 1 = *never* to 5 = *often*, was chosen as appropriate response format for these items.

##### Refinement of the initial item pool

The resulting 30-item pool was examined by a second group of three experts, composed of one psychologist, one psychoanalyst and one social methodologist who were asked to independently rate the relevance to the construct of each item on a 1 to 5 Likert scale (1 = *strongly disagree*, 5 = *strongly agree*). The psychoanalyst and the psychologist expert of the health services set up for violence against women evaluated the relevance of the emerged item pool with theoretical principles and with studied phenomenology in order to guarantee the content validity of the instrument. The methodologist, instead, rated the degree of adherence to the response format and the formulation of the items according to the criteria of brevity, simplicity, exclusion of possible linguistic ambiguities. Based on the collected rating, the 30-item pool underwent syntactic changes and reformulations, which led to a reduction in the number of items. Two items were deleted; one, related to the area of the affect of fear, was evaluated redundant, and another, belonging to the area of the affect of guilt, resulted ambiguous. Furthermore, linguistic ambiguities, double statements, multiple negations and redundant frequency adverbs in the response format were deleted and some changes were made to the instructions and to the terminology.

At the end of this selection, 28 items were retained and grouped as follows: 5 related to fear, 7 related to terror, 10 related to shame, and 6 related to guilt.

The 28-item pool was submitted to a further screening aimed at examining its comprehensibility. It was administered to the same group of 10 women who had participated in the qualitative study described above, since it was considered as a representative sample of the population under examination. This preliminary administration confirmed the comprehensibility of the item and, therefore, did not result in any changes.

All aspects of the study involved the informed consent of each participant, according to the ethical guidelines of Helsinki Declaration. Moreover, participants were informed about the confidentiality of their responses and their anonymous treatment. Participants read a web page with the informed consent document before starting the online survey. The online consent form containing all the required elements, consisting of purpose of the research, nature of participation, description of research procedures, description of risks, voluntariness of participation, right to withdraw at any time without penalty, handling of data (anonymity and confidentiality), contact information for researcher, and contact information for concerns about the project were read and submitted by clicking a button below the text to consent to participate to the survey. There was no honorarium for completing the assessment.

The protocol was approved by the ethics committee of Section of Psychology and Education Sciences, University of Naples Federico II, Italy.

### Statistical Analysis

A split-sample cross-validation procedure (Bollen, 1989) was performed on our sample. Data from subsample A and subsample B were respectively subjected to an exploratory factor analysis (EFA- study 1), and to a confirmatory factorial analysis (CFA- study 2), based on the factor structure derived from the exploratory analysis. Model fit was measured by means of the following fit indexes that are suggested as most important (Hu and Bentler, 1998, 1999; MacCallum and Austin, 2000): (a) the chi-square (χ^2^) statistic and its degrees of freedom; (b) the Tucker–Lewis Index (TLI); (c) the comparative fit index (CFI); (d) the root-mean-square error of approximation (RMSEA) and its 90% confidence interval (CI); and (e) the standardized root mean square residual (SRMR). According to Schermelleh-Engel et al. (2003), the model fit the data when: χ^2^/df < 2, CFI and TLI > 0.97, SRMR < 0.05, and RMSEA < 0.05 (90% CI: the lower boundary of the CI should contain zero for exact fit and be <0.05 for close fit), in any case also values between 0.05 and 0.08 were considered by some authors as indicatory of a good suitability of the model (Browne and Cudeck, 1993; Hu and Bentler, 1999).

Cronbach’s alpha and correlations have been used to assess descriptive statistics and internal consistency.

## Results

### Study 1

#### Subsample A

The sample included 151 participants (age *M*: 30.23; *SD*: 8.87 years). As regards their marital status, 83.4% of the women was unmarried, 10.6% married, 2.6% divorced, 2% separated, and 1.3% through a divorce. As regards the sexual orientation 83.4% of the women were heterosexual, 5.3% bisexual and 1.3% homosexual. Regarding level of education: 39.1% had a master’s degree, 21.2% a 3- year degree and 21.3% a high-school diploma and 16.7% a post- graduate degree. As regards the employment 29.8% of subjects were students, 25.2% were self- employed workers, 14.6% were employed and 4.6% were unemployed. Moreover, 68.9% of study participants were involved in a current romantic relationship, 88.1% had no children and the remaining had from one to three offspring.

##### Exploratory factor analysis (EFA)

The structure of the VITA Scale has been evaluated through a series of EFA using the principal axis factoring (PFA) method in subsample A. PFA has been chosen because of its capacity to recover weak factors and be fairer than principal component analysis (Widaman, 1993) especially when working on small samples (Briggs and MacCallum, 2003). Firstly, it has been tested a one-factor model where all the VITA Scale items were free to load on a single latent component. The one-factor solution explained the 37.54% of the total variance, with eigenvalue equal to 11.05. Absolute factor loadings for each item were greater than 0.30, except for the items (#5) “Ho reagito alla paura chiedendo aiuto” (“I reacted to the fear by asking for help”) and (#3) “L’agitazione mi ha spinto a reagire” (“Agitation pushed me to react”). However, a careful inspection of the scree test (Cattell, 1966) and the inclusion of the factor with eigenvalues > 1 (Kaiser, 1960) suggested the extraction of four or five factor latent components. Based on the previous results, a second PFA was performed extracting five factors with Direct Oblimin rotation. Despite the solution accounting for more than 60% of the total variance, several double factor loadings (>0.30) were observed in the pattern structure. Again, the fifth factor resulted to be composed of a single item “Mi sento/sentivo sporca” (#20) (“I fee/felt dirty”). Therefore, the five-factor model tested could not retained as a reliable solution, both from the statistical and theoretical examination. The last model tested the presence of four factor latent components. Following the authors construct theory of the VITA Scale, a four-factor solution was extracted using PFA with Direct Oblimin rotation. The Kaiser-Meyer-Olkin index was 0.900 suggesting an appropriate measure of sampling adequacy (Tabachnick and Fidell, 2007). The significant Bartlett’s Test of Sphericity (2767,990; gdl = 153; *p* = 0.001) suggested the adequacy of sample to the EFA. According to the scree test (Cattell, 1966), four factors could be extracted, accounting for the 63.96% of the total variance. All the VITA Scale items showed absolute loadings for each item greater than 0.30 (see **Table 1**). Only six items showed secondary loadings (#2, #10, #13, #15, #18, and #25). Based on the content analysis, nine items (from #13 to #22, without #18) loaded on the first factor called “Shame”; five items (from #1 to #5) loaded on the second factor called “Fear”; six items (#from #23 to #28) loaded on “Guilt” factor; and, eight items (from #6 to #12 and item #18) loaded on factor defined “Terror.”

**Table 1:** Exploratory factor analysis (EFA) loadings performed on subsample A (*N* = 151).

#Item	Original dimension	Item	Factor			
			1	2	3	4
19	Shame	VITA ITEM 19	0,960			
16	Shame	VITA ITEM 16	0,911			
21	Shame	VITA ITEM 21	0,619			
14	Shame	VITA ITEM 14	0,619			
20	Shame	VITA ITEM 20	0,591			
17	Shame	VITA ITEM 17	0,536			
22	Shame	VITA ITEM 22	0,532			
15	Shame	VITA ITEM 15	*0,534*		*0,351*	
13	Shame	VITA ITEM 13	*0,360*		*0,326*	
3	Fear	VITA ITEM 3		0,727		
1	Fear	VITA ITEM 1		0,689		
5	Fear	VITA ITEM 5		0,627		
4	Fear	VITA ITEM 4		0,479		
2	Fear	VITA ITEM 2		*0,551*		*-0,409*
24	Guilt	VITA ITEM 24			0,910	
26	Guilt	VITA ITEM 26			0,737	
27	Guilt	VITA ITEM 27			0,717	
28	Guilt	VITA ITEM 28			0,706	
23	Guilt	VITA ITEM 23			0,602	
25	Guilt	VITA ITEM 25			*0,396*	*-0,338*
8	Terror	VITA ITEM 8				-0,821
9	Terror	VITA ITEM 9				-0,811
12	Terror	VITA ITEM 12				-0,802
7	Terror	VITA ITEM 7				-0,768
6	Terror	VITA ITEM 6				-0,600
11	Terror	VITA ITEM 11				-0,591
18	Shame	VITA ITEM 18	*0,386*			*-0,447*
10	Terror	VITA ITEM 10		*0,317*		*-0,317*

		Eigenvalues	10,678	3,267	1,393	0,968
		% explainedvariance	38,134	11,67	4,975	3,458

*In Italic double item loadings*.

### Study 2

#### Subsample B

The sample included 151 participants (*M*: 30.53; *SD*: 23.7 years). 79.5% of the women was unmarried, 14.3% married, 0.7% divorced, 5.3% separated. 90.7% of the women are heterosexual, 6% bisexual, 3.3% homosexual. Regarding level of education: 40.4% had a high-school diploma 22.5% had a master’s degree, 18.5% a 3- year degree and 17.2% a post- graduate degree. 41.7% of the women were female students, 27.2% was self-employed workers, 6.6% were without any employment, 13.2% were employed.

74.2% were in romantic relationship, 84.1% had no children and the remaining had from 1 to 3 offspring.

##### Confirmatory factor analysis (CFA)

A CFA (Bollen, 1989) was carry out using the MPLUS 7 statistical package (Muthén and Muthén, 2012) on the subsample B. Descriptive statistics for the Subsample B revealed no missing values and several departures from the normality of the data. Specifically, item #10 showed skewness and kurtosis values that exceed the cut-off criteria of ±3 (Curran et al., 1996).

Due to the asymmetrical distribution of data, the responses to the VITA Scale items should be better evaluated at the categorical rather than the metric level. However, the robust unweighted least squares (ULSMW) method using a diagonal weight matrix and robust standard errors and a mean- and variance adjusted χ^2^ test statistic (Muthén and Muthén, 1998, 2004) was used to estimate parameters. Like WLSMV, the ULSMV estimator were more likely to catch small structural links with precision when data was slightly or moderately asymmetric, and when small sample sizes were used.

The one-factor and the four-factor refined models (without items with double loadings) emerged from the previous EFA, versus the four-factor model, that followed authors’ theoretical assumptions (Troisi, 2017), were tested. The unstable five-factor model was excluded from the comparison, based on the results from the previous EFA.

As seen in **Table 2**, the four-factor model fit the data slightly better than the refined four-factor model and the one-factor model. Specifically, the one-factor model fitted the data worst. All the chi-squared values were significant (*p* < 0.001), and the ratio χ^2^/df indicated a slightly better fit of the four-factor model (χ^2^ = 540.789; χ^2^/df = 1.57) than the refined model (χ^2^ = 355.389; χ^2^/df = 1.75). Likewise, CFI (four-factor, 0.970; refined model, 0.960) and RMSEA (four-factor, 0.062; refined model, 0.071) indices confirmed the better fit of the four-factor model. The TLI index of the two models showed no difference. TLI and CFI were all above 0.97 and SRMR was close to 0.05 (Schermelleh-Engel et al., 2003), indicating a close fit of both models to the empirical data. These results showed that the exclusion of the items with EFA double loadings (#2, #10, #13, #15, #18, and #25) did not contribute to improving the model.

**Table 2:** Confirmatory factorial analysis (CFA) indices performed on the subsample B.

Models	χ^2^	*df*	χ^2/^*^df^*	TLI	CFI	RMSEA	90% CI
One factor	704.995	350	2,01	0.939	0.943	0.082	0.073 0.091
Four factors	540.789	344	1,57	0.966	0.970	0.062	0.051 0.071
Four factors refined^∗^	355.389	203	1,75	0.965	0.960	0.071	0.058 0.083

*df, degrees of freedom; TLI, Tucker–Lewis Index; CFI, Comparative Fit Index; RMSEA, root-mean-square error of approximation; 90% CI, 90% confidence interval of RMSEA; SRMR, standardized root mean square residual*.

^∗^*Four factor refined model without EFA double loadings items #2, #10, #13, #15, #18, and #25*.

**FIGURE 1:**
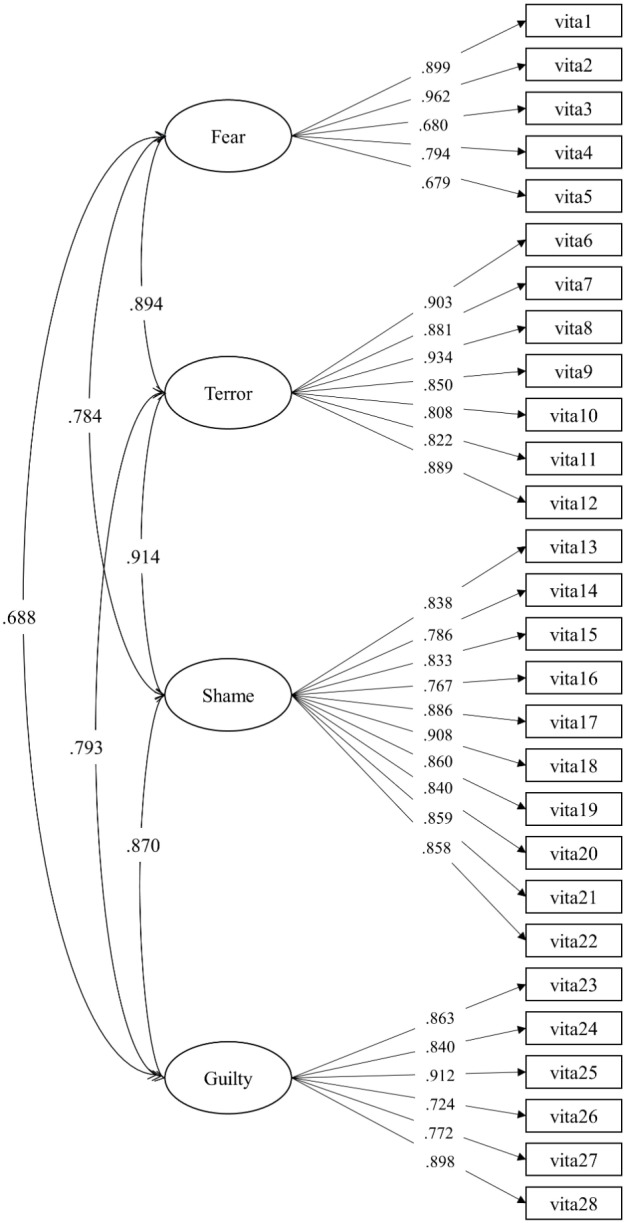
Confirmatory factorial analysis (CFA) standardized estimate for the four-factor model of the VISTA scale (subsample B, *N* = 151).

In **Figure 1** the standardized factor loadings of the four-factor CFA model was shown, as well as the path coefficients, among the four latent factors. All the items loaded considerably (>0.75) on the respective factors, and all the four latent factors highly correlated (from 0.68 to 0.89).

##### Construct validation

Pearson correlational analyses were used to explore the associations between the VITA subscales and other related measures (**Table 3**).

**Table 3:** Pearson correlations between VITA and external measures and descriptive statistics.

VITA subscale						Descriptive statistics		
	Shame	Guilt	Terror	Fear	Total scale	a	*M*	*SD*
DERS	0.242**	0.315**	0.303**	0.043	0.252**	0.86	80.95	22.25
Non-accept	0.338**	0.313**	0.305**	0.062	0.346**	0.89	14.91	6.20
Goals	0.401**	0.285**	0.386**	0.117	0.346**	0.85	5.54	2.44
Impulse	0.262**	0.187*	0.252**	0.097	0.202*	0.85	14.72	5.56
Awareness	-0.132	-0.059	-0.041	-0.097	-0.096	0.81	11.20	4.2
Strategies	0.239**	0.158	0.194*	-0.025	-0.066	0.82	15.63	4.74
Clarity	-0.004	-0.75	0.004	-0.021	0.147	0.84	19.11	6.36
IES	0.550**	0.328**	0.534**	0.327**	0.575**	0.93	34.24	12.6
Intrusion	0.510**	0.349**	0.508**	0.294**	0.544**	0.92	15.76	6.61
Avoidance	0.499**	0.258**	0.486**	0.324**	0.520**	0.89	18.27	7.11
OAS	0.349**	0.461**	0.331**	0.120	0.382**	0.94	41.44	16.19
Inferiority	0.317**	0.435**	0.284**	0.077	0.332**	0.92	17.83	7.93
Emptiness	0.292**	0.334**	0.315**	0.143	0.337**	0.83	8.67	4.13
Mistake	0.327**	0.450**	0.314**	0.128	0.367**	0.86	14.95	5.80
COPE-NVI 25	0.144	0.232**	0.122	0.122	0.144	0.85	86.38	17.19
Avoidance strategies	0.228**	0.267**	0.232**	0.059	0.231**	0.76	12.84	5.056
Transcendent orientation	-0.005	0.041	-0.030	-0.076	-0.037	0.96	8.55	6.17
Positive attitude	0.178*	0.231**	0.102	0.096	0.206*	0.80	24.40	6.193
Social support	0.054	0.062	-0.020	0.070	-0.034	0.94	20.46	6.47
Problem orientation	0.035	0.027	0.091	0.204*	0.046	0.83	20.08	5.41
TDI	0.348**	0.365**	0.202	0.062	0.280*	0.95	40.72	16.13

^∗∗^*p < 0.01; ^∗^*p* < 0.05*.

The subscales of the VITA Scale *Shame* and *Guilt* were positively correlated with the DERS subscales *Non-acceptance*, *Goals* and *Impulse*, with both subscales of the IES (*Intrusion* and *Avoidance*) and with all subscales of the OAS (*inferiority*, *emptiness*, and *mistake*). Furthermore, the subscale of Shame and Guilt were positively correlated with the subscale of COPE-NVI 25 that were *Avoidance Strategies* and *Positive Attitude* and with the total score of the TDI. The VITA subscale of *Shame* was positively correlated with the DERS subscale of *Strategies.*

The VITA subscale of *Terror* was positively correlated with the DERS subscales of *Non-acceptance*, *Goals* and *Impulse* and *Strategies*, with both the subscales of IES (*Intrusion* and *Avoidance*), with all subscales of OAS and with the subscale of COPE-NVI 25 related to *Avoidance Strategies*.

VITA-Scale *Fear* was positively correlated with both the IES subscales (*Intrusion* and *Avoidance*) and positively correlated with the COPE-NVI 25 subscale of *Problem Orientation*.

Significant correlations were found between the total score of the VITA and the total scores the used concurrent measures, that were OAS, IES, DERS (*p* < 0.01) and TDI (*p* < 0.05).

## Discussion

Intimate partner violence is the most common form of violence against women (Campbell, 2002). Unfortunately, it is also the most difficult form of violence to recognize (Herman-Lewis, 1992; Hirigoyen, 2005).

The results of the current study widely confirmed the main role played by four affects of terror, fear, guilt and shame, in situation of IPV in line with the authors’ theoretical assumptions (Margherita and Troisi, 2014; Margherita et al., 2014; Nunziante Cesàro and Troisi, 2016).

Particularly, this research confirmed the main role, within the women’s subjective affective experience of victimization, of the affect of shame (Follingstad et al., 1991; Sippel and Marshall, 2011; Shorey et al., 2011), of guilt (Beck et al., 2011), and of fear (Kilpatrick et al., 1989; Riggs et al., 1992; Weaver and Clum, 1995; Scheffer Lindgren and Renck, 2008).

Summing up, the current study aimed at measuring the variety and the complexity of the post-traumatic affectivity of the women suffered from IPV experienced through a specific and newly developed instrument, named VITA Scale (Intimate Violence and Traumatic Affects Scale).

This scale showed a clear factor structure and strong psychometric properties in a sample composed of women suffered from IPV.

Reliability analysis indicated that the VITA Scale, as well its subscales related to the different affects, showed an excellent Cronbach alpha value. The EFA and CFA showed a fully satisfactory fit. The dimensions emerging from these analyses were in line with theoretical expectations (Margherita and Troisi, 2014; Margherita et al., 2014; Nunziante Cesàro and Troisi, 2016).

Results of correlational analysis was in line with the theoretical expectations: the intensity of post-traumatic affectivity was positively and significantly correlated with external shame, the intensity of depression, the impact of trauma, the affective dysregulation and the lack of strategies and it is not related to the implementation of coping skills. The VITA subscale of *terror* was positively correlated with the subscale *avoidance strategy* of the COPE-INV25 and the subscale of *fear* was positively correlated with the *Problem Orientation* subscale of the COPE-NVI. These last two correlations showed that fear was more associated with the possibility of facing the problem in an “activity” dimension, while terror was more associated with a “passive” avoidance response.

Particular attention should be devoted to the distinction of the affects of terror and fear. While fear assumes a more protective function, terror is denoted as a psychic state, more intense than fear: it emerges when facing with a threat of extreme danger, that could be real or fictitious and would lead to a state of passivity (Clit, 2002; Nunziante Cesàro and Troisi, 2016; Troisi, 2017).

Previous studies, not making a specific distinction between fear and terror, considered fear in the IPV resulting from both the perceived risk of violence and the uncontrollability of this risk (Smith et al., 1995). The distinction between fear and terror, as proposed in the current study, is also supported by neurophysiological studies, which underlined that neuronal circuits of the amygdala, the hypothalamus and the periaqueductal gray substance had sub-zones distinguished for active defenses, such as attack-escape, and for passive ones, such as freezing (Hagenaars et al., 2014).

In the face of danger, the emergence of the affects of terror and shame shows itself as the first defense to maintain the psychic equilibrium, a first form of protection against the disorganization induced by the trauma. If, in dealing with the malaise, the possible actions are no longer adaptive, these affects can come into play with the loss of the feeling of Self.

There are, for example, two different levels of shame: a *toxic shame*, associated with a sense of constriction, anger and withdrawal, and with an intolerable punitive isolation, and on the other level a *humanizing shame* that sharpens empathy. This is a shame that has been given recognition either by others or by themselves or by both (Kilborne, 2002).

Feeling shame is the first sign of subjectivation, of being still a subject among subjects, this affect besides being first human feeling, it is a social feeling at the limit between intra and inter-subjective (Barazer, 2000).

Several studies have shown that the affect of shame is associated with the maintenance of PTSD symptoms over time (Andrews et al., 2000).

In the guilt proneness of women who have suffered from violence, not specifically psychoanalytic approaches identify a tendency to feel regrets or remorse for past behavior judged as wrong, while shame refers more to a lowering of self-esteem and emptiness feelings (Gilbert, 2000) and sense of inferiority to the other (Tangney, 1996; Tangney and Dearing, 2002), and is considered a less adaptive affects than the guilt.

Some other studies have questioned whether shame could be a predisposing factor, rather than a consequence of IPV (Harper and Arias, 2004).

Working with such affects, in situations of violence, can be useful in processing the trauma, in order to return to the victim that functional role for the psychic life of the individual that allows a subject to became aware of his/her own internal world and to inscribe the traumatic experience in temporality (Herman-Lewis, 1992; Bohleber, 2007; Levine, 2014).

It is necessary that these affects can emerge to be recognized and elaborated, to limit the disruptive effects of the trauma and reconstruct the event by placing it in a space and in a time and to increase the woman’s ability to think and elaborate those affective experiences that escape any attempt of nomination.

Also, in line with this assumption, the scale of post-traumatic affects here built (VITA Scale) has foreseen the use of metaphor, which stands as a mediator between unspeakable affections and representations (Tisseron, 1992).

It is important to underline that the traumatic experience of the women who suffered from IPV differs from traumas after another stressful event of life. Trauma after IPV is an interpersonal trauma, caused by another person. This type of trauma, whose nature is relational and lasting, is often a “complex trauma” (Herman-Lewis, 1992), whereas the traumatic experience is not a single event but it is repeated and prolonged. This specific situation can make the nervous system reactive, as if in a constant state of alarm and has a higher PTSD risk than other types of trauma, such as trauma associated with natural disasters (Kessler et al., 2017).

The VITA Scale could be a useful tool for the clinician to investigate the affective state of the woman at the time of access to services, to assess the awareness of the woman in her internal world after the trauma, in order to better address the clinical practice and therapeutic intervention planning. Moreover, the use of the instrument could facilitate the recognition of the affects that emerged in the woman following the traumatic experience. This tool could also be useful to broaden the scientific knowledge on the subjective affective experience of victimization, for which several studies have emphasized the need (Harper and Arias, 2004) and for recognition of change in the therapeutic process (Halfon et al., 2016). Furthermore the VITA Scale may be helpful in investigating the role played by affects in different situations of violence.

The treatment of the traumatized woman requires specific clinical work aimed at developing the ability to process traumatic affects and only an adequate tuning with the precise affective states can support the therapeutic alliance and reconstruct the sense of security threatened by the traumatic event (Caretti et al., 2013; De Luca Picione et al., 2017, 2018).

This study presented several limitations. The sample recruited online is not actually discriminant of a clinical sample (e.g., Balsamo et al., 2013b). Furthermore, the validation study was not aimed at identifying the effectiveness of the tool in monitoring the therapeutic intervention process and in understanding the specificities that these affects take on in relation to the type of specific violence suffered. Another limitation consists in the fact that the explored affectivity may not be exhaustive of the complexity of the women’s emotional reaction following trauma. Future research needs to confirm the results on a clinical sample and to measure whether this instrument is sensitive to changes in the therapeutic process with women who have suffered from IPV. Future directions will be addressed to a more in-depth exploration of the consequences of violence for women’s emotional experience to refine the content validity of the scale.

## Author Contributions

The author contributes at the whole article, in each of its part.

## Conflict of Interest Statement

The author declares that the research was conducted in the absence of any commercial or financial relationships that could be construed as a potential conflict of interest.
